# Review of recent progress in the development of electrolytes for Cd/Pb-based quantum dot-sensitized solar cells: performance and stability

**DOI:** 10.1039/d4ra01030b

**Published:** 2024-05-20

**Authors:** Bayisa Batu Kasaye, Megersa Wodajo Shura, Solomon Tiruneh Dibaba

**Affiliations:** a Department of Applied Physics, School of Natural and Applied Sciences, Adama Science and Technology University Adama Oromia Ethiopia megersawodajo@gmail.com

## Abstract

Quantum dot-sensitized solar cells (QDSSCs) represent an exciting advancement in third-generation photovoltaic solar cells owing to their ability to generate multiple electron–hole pairs per photon, high stability under light and moisture exposure, and flexibility in size and composition tuning. Although these cells have achieved power conversion efficiencies exceeding 15%, there remains a challenge in enhancing both their efficiency and stability for practical large-scale applications. Therefore, in this review, we aimed to investigate recent progress in improving the long-term stability, analyzing the impact of advanced quantum dot properties on charge-transport optimization, and assessing the role of interface engineering in reducing recombination losses to maximize QDSSC performance and stability. Additionally, this review delves into key elements such as the electrolyte composition, ionic conductivity, and compatibility with counter electrodes and photoanodes to understand their influence on power conversion efficiencies and stability. Finally, potential directions for advancing QDSC development in future are discussed to provide insights into the obstacles and opportunities for achieving high-efficiency QDSSCs.

## Abstract 2

QDSSCs owing the ability to generate multiple electron–hole pairs per photon, high stability under light and moisture exposure, and flexibility in size and composition tuning.

## Graphical abstract

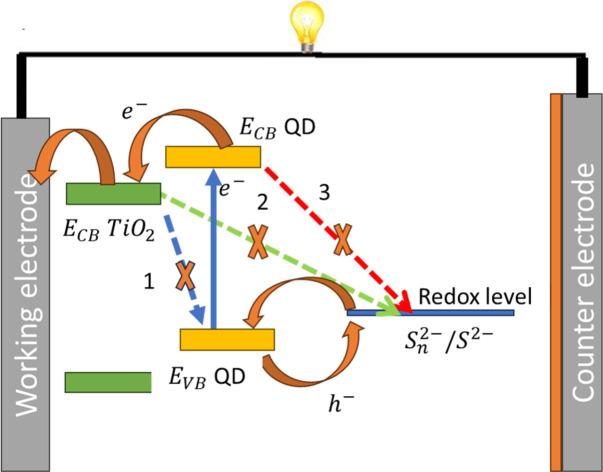

## 1. Introduction

Fossil fuels are non-renewable sources of energy that causes toxic emissions, global warming, energy crises, and environmental pollution. Therefore, fossil fuels must be replaced with renewable energy sources such as solar cells, wind energy and hydroelectric power.^1,2^ Among these, solar cells use sunlight radiation to generate electricity. Based on both the material used and level of economic maturity, solar cells are often divided into three generations, namely, silicon-based materials (first generation), thin film solar cells (second generation), and third-generation solar cells (low cost and high efficiency). Compared to the first- and second-generation solar cells, the third-generation solar cells are characterized by low cost, high efficiency, environmentally friendly, transparent, and plastic substrates.^3^ Among the quantum dot-based solar cells, quantum dot-sensitized solar cells (QDSSCs) harvest light using nanostructured quantum dots.^4^ Quantum dots are unique in their optical, electronic, and chemical properties such as tuneable band gap, multiple exciton generation, and high absorption coefficients.^5^

**Fig. 1:**
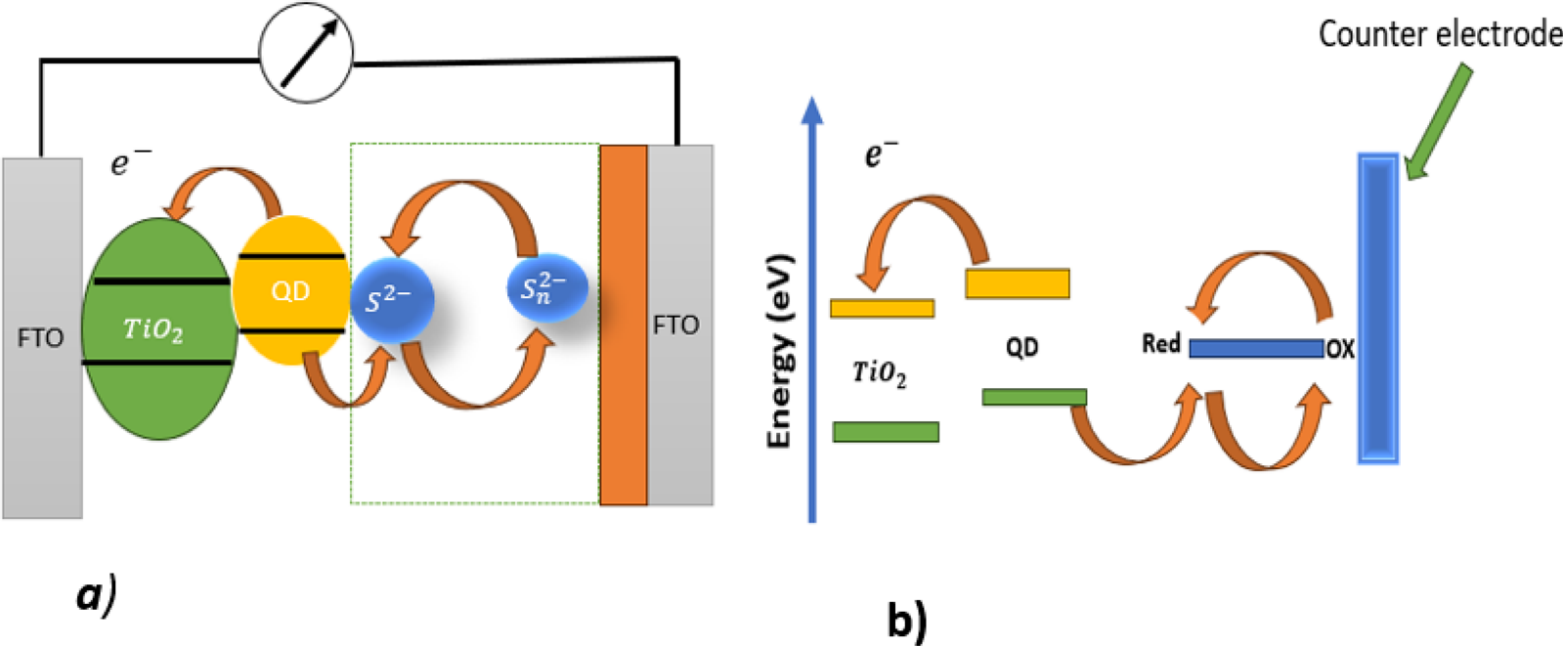
(a) Diagram of a quantum dot-sensitized solar cell; (b) energy level diagram for a quantum dot-sensitized solar cell.

A QDSSC works by absorbing photons using quantum dots as absorbers. The QDs absorb photons, then electrons are excited from the ground state (VB) to higher energy (CB) states when the photon energy exceeds the bandgap of the QD. Electron–hole pairs are formed as a result of Coulomb attraction between opposite charges. Because of the thermal energy exceeds the binding energy, these electron–hole pairs dissociate. Then electrons are injected into the conduction band of the wide band gap of the semiconductor, and diffuse toward the counter electrode through an external circuit. As the holes moves toward the electrolyte, the oxidation species diffuse toward the counter electrode and are reduced by receiving electrons from the external circuit.^6^ To increase electron injection into a wide semiconductor, the conduction band (CB) of the wide semiconductor must be lower than that of a quantum dot.^7^ A typical QDSSC device structure and its working principles are shown in Fig. 1a, and an energy level diagram of a quantum dot sensitized solar cell is depicted in Fig. 1b.

For a QDSSC to be highly efficient, the QD must have a high conduction band edge, wide absorption range, and large QD number (loading amount onto a wide band gap). QDSSCs consist of a working electrode (*e.g.* TiO_2_ or Zano) counter electrode (*e.g.* rGO or CuS), and electrolyte (*e.g.* polysulfide).

A counter electrode receives electrons from an external circuit and promotes them to the electrolyte to catalyze the reduction reaction of the oxidized electrolyte at the interface between the counter electrode and electrolyte. For QDSSCs, high conductivity, high catalytic activity, and excellent corrosion resistance are crucial. However, QDSSCs based on platinum electrodes exhibit low power conversion because the polysulfide electrolyte reacts with platinum, in contrast to other materials such as CoS, CuS, and NiS,^8–11^ or combinations of carbon compounds with other compounds, such as graphene hydrogel/CuS or graphene/PbS.^12^

In recent years, the use of carbon materials and composites has increased. As a result of their excellent catalytic activity towards polysulfide redox couples, the use of such materials has enabled QDSSC to become more efficient.

Electrolytes are also crucial for QDSSCs. The electrolytes in QDSSCs are responsible for regenerating oxidized quantum dots after the electrons are injected into the semiconductor, transporting the positive charge to the counter electrode, and transferring the charge efficiently between the anode and counter electrolyte. QDSSCs require electrolytes with high solubility, high ion mobility, and fast electron-transfer kinetics.

QDSSCs have been investigated using several electrolytes, including liquid electrolytes, which exhibit higher performance but lower stability. In contrast, quasi-solid-state and solid-state electrolytes have higher stability but lower power conversion than liquids. This review paper focuses on strategies to improve the stability and performance of quantum dot-sensitized solar cells as a result of electrolyte improvements.

## 2. Electrolytes

High corrosive resistance, good redox potential for regeneration of the oxidized quantum dots, and high ionic conductivity for hole transfer^10^ are crucial requirements for an electrolyte in a solar cell. The electrolyte is located between the cathode and anode to exchange charge carriers or separate the positive and negative electrodes to maintain the long-term stability of QDSSCs.

The use of highly wettable solvents, with low surface tensions and low dielectric constants make nanostructured films highly permeable. Ethanol, methanol, alcohol, and water are solvents with high wettability, low surface tension, and low dielectric constant, respectively. Generally, solvents with low surface tension exhibit high wettability.^13^

In general, to improve the performance of QDSSCs, it is important that electrolytes are dissolved in solvents that are highly wettable, and highly permeable to promote redox *versus* diffusion and reduce the charge-recombination kinetics.^14,15^ However, low-surface-tension solvents have low ion-dissociation capabilities, and instead, the use of high-surface-tension and low-surface-tension co-solvents is advantageous.^16^ Based on the phase, there are three possible electrolytes: liquid, quasi-solid-state, and solid electrolytes.

An electrolyte in a QDSSC works based on eqn (1) and (2).

The hole moves from the oxidized QD to the electrolyte, then the electrolyte is oxidized:^16^

1 S^2−^ + 2h^+^ → S

An oxidized electrolyte accepts electrons from the counter electrode and is reduced:

2 S_*x*_^2−^ + 2e^−^ → S_*x*−1_^2−^ + S^2−^

### 2.1 Liquid electrolytes

As conductive pathways for photovoltaic redox reactions, liquid electrolytes in QDSSCs contain oxidizing and reducing components in an aqueous or organic medium (solvent). Due to their non corrosive and non-photodegradation properties, polysulfide electrolytes (containing Na_2_S and S) can be corrosive and non-photodegradation properties, polysulfide electrolytes (containing Na_2_S and S) can be used.^17^ Despite their high redox potential, such electrolytes reduce the open circuit voltage and have a poor fill factor, which reduces their power conversion efficiency (PCE).^18^ The performance of the device can deteriorate due to oxygen permeation and volatility. Liquid electrolyte cations adsorb strongly on nanoporous photoanodes with a downshift of the TiO_2_ conduction band (CB) and a decrease in the open circuit voltage. To reduce charge recombination at the photoanode and at the electrolyte interface, as well as at the electrolyte and counter electrode interface, several additives are used.

**Fig. 2:**
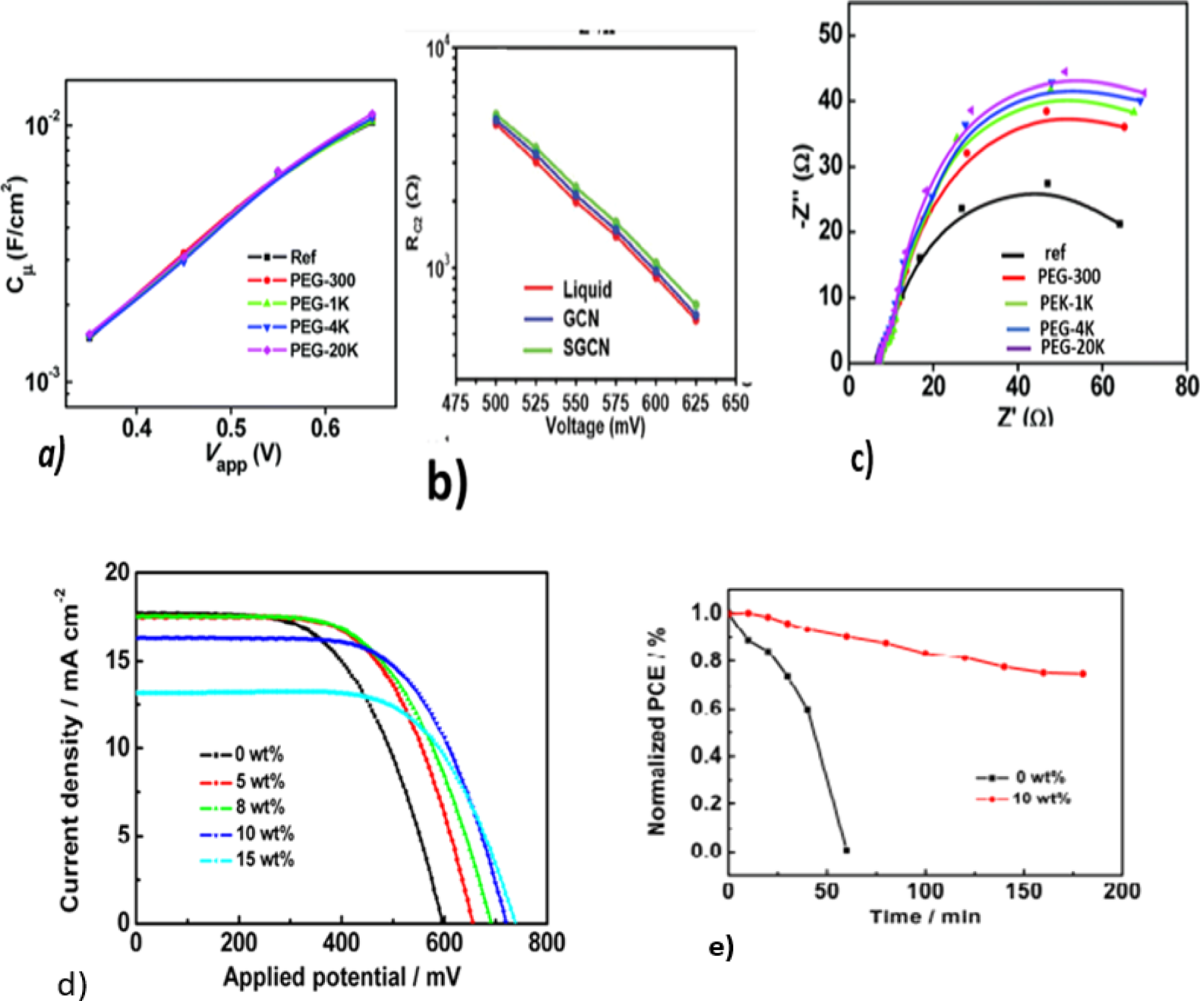
EIS characterizations of ZCISe QDSCs with electrolytes containing different PEG additives. (a) Chemical capacitance. Reprinted from ref. 15 with permission from the Royal Society of Chemistry, copyright 2018; (b) recombination resistance. Reprinted from ref. 18 with permission from the Royal Society of Chemistry, copyright 2021; (c) Nyquist plots under a forward bias of −0.65 V; (d) *J*–*V* curves for CdS/CdSe co-sensitized QDSCs with different electrolyte contents. Reprinted from ref. 15 and 24; (e) stability tests of a QDSSC fabricated with a polysulfide electrolyte. Reprinted from ref. 24.

In polysulfide electrolytes, additives are adsorbed onto wide band gaps and form molecular complexes that block the surface trap states of photoanodes.^15,19^ This increases the short circuit current, open circuit voltage, and power conversion efficiency of QDSSCs. The use of polyethylene glycol (PEG) as a polymer additive to modify the polysulfide electrolyte in a ZCISe QDSSC reduced charge recombination and enabled achieving a PCE of 10.20%.^15^ PEG molecules containing hydroxyl and oxygen atoms coordinate strongly with TiO_2_. To prevent charge recombination, these molecules are used as a passivation layer. Reportedly, some additives, like PEG,^20^ TEOS,^19^ and PVP,^21^ in the polysulfide electrolyte solution do not change the CB edge or the electron density of TiO_2_, as depicted in Fig. 2a. These additives do not change the energy level of TiO_2_, rather they act as a coating or layer to improve its stability by reducing electron recombination at the photoanode/electrolyte interface. The effect can be strengthened by increasing the concentration of the additive, as shown in Fig. 2b. It can be seen that as the Mw of the PEG additives in the electrolyte increases, the diameter of EIS semicircles increases systematically (Fig. 2c).

Meng and colleagues modified polysulfide electrolytes with fumed SiO_2_ nanoparticles to reduce electron recombination and increase the electron lifetime in a CdSeTe QDSSC. Silica nanoparticles were dispersed using liquid polysulfide to achieve a PCE of 11.23%. Gels were formed when silica nanoparticles were dispersed in polysulfide and formed hydrogen bonds with water molecules silanol (Si–OH),^22^ which was hydrolyzed under high pH conditions, leading to a degradation of the silica nanoparticles and ultimately the degradation of the device.^23^ Additionally, the insertion of silica nanoparticles into a polysulfide electrolyte requires additional fabrication steps and increases the fabrication cost.

With NH_2_-rich silica nanoparticles (A-SiO_2_) as an additive, Pin Ma's group produced stable ionic-liquid gel-based electrolytes with an improved PCE of 7.11% for CdS/CdSe QDSSCs, and the stability of the device was improved through the use of additives,^24^ as shown in Fig. 2d and e, respectively. NH_2_-rich silica nanoparticles, also called amino-functionalized silica nanoparticles, have also been tested.^25^ These NH_2_ amino group, however, can affect the electrolyte additives, since it can accept (H^+^) from water and form NH^4+^ and OH^−^. Hydroxide ions can form strong bases that can degrade silica nanoparticles, which in turn will reduce device stability.

Beiraghdar's group modified a polysulfide electrolyte using different additives, such as amine, thiourea, and urea, in CdS/CdSe QDSSCs, and reported a best PCE of 3.42% using a thiourea additive. By adding thiourea to liquid polysulfide, electrons are transferred to TiO_2_ through the amine groups and sulfur atoms, resulting in a longer electron lifetime.^26^ However, at high temperatures and UV exposure, amines may undergo degradation, resulting in cell component degradation and reduced performance.^23^

According to Rasal, Akash S. *et al.*, the addition of graphitic carbon nitride (GCN) and sulfur-rich graphitic carbon nitride (SGCN) into the polysulfide electrolyte enhanced the PCE of CIS QDs as sensitizers in QDSSCs with liquid, GCN, and SGCN contents of 6.16%, 6.73% and 7.13%, respectively. Additionally, the charge-recombination resistance between the TiO_2_/QDs/electrolyte interfaces was reduced by adding the additive,^18^ resulting in a longer electron lifetime.

Table 1 summarizes the results from studies on the solar cell parameters of QDSSCs based on liquid electrolytes.

**Table 1:** Solar cell parameters of QDSCs based on liquid polysulfide electrolytes^a^

QD	Electrolyte	CE	*J*_sc_	*V*_oc_	FF	*η*%	Ref.
CdSe	Na_2_S, S, KCl	NPCN	7.51	0.71	0.50	2.7	27
CdS	Na_2_S, S, NaOH	Cu_2_S/CP	10.52	0.606	05.57	2.85	25 and 28
CdSe	Na_2_S, S	Pt	7.51	0.71	0.50	2.7	29
CdSeTe	Na_2_S, S, KCl	GH/CuS	20.69	0.786	0.66	10.71	12
CdS/CdSe/ZnS	Na_2_S, 2S	g-C_3_N_4_/CuS	0.63	19.35	30.17	3.65	30
CdS	Na_2_S, S, KCl	CB/CuS	0.43	6.36	47	1.3	31
PbS/CdS–C/S	Na_2_S, S, KCl	Cu_2_S	627.7	14.85	43.90	4.16	32
CdSeTe-	Na_2_S, S, KCl	CuS	638	13.90	0.51	4.60	33
ZCISe	Na_2_S, S, KCl	Cu@CNR	0.628	26.46	0.593	9.50	34
CdSeTe/ZnS	Na_2_S, S	Cu_2−*x*_S	20.78	0.702	0.636	9.28	35
CdS	Na_2_S, S	PbS	0.59	8.59	0.58	2.91	36
CdS	Na_2_S, S, KCl	Cu_2_S-SiW_12_/MoS_2_	15.78	0.622	0.431	4.28	37
CdSe	Na_2_S, S, KCl	Au	502.6	11.66	0.49	2.9	38
CdS_*x*_Se_1−*x*_	Na_2_S, S	Cu_2_S	12.5	0.57	0.55	3.69	39
CdSeTe	Na_2_S, S, KCl	CNT@rGO@MoCuSe	0.633	20.54	0.636	8.28	40
CdSeTe	Na_2_S, S KCl	Cu_*x*_Se	0.654	20.82	60.13	8.72	41
Zn–Cu–In–Se	Na_2_S, S	CNT/GH/CuS	0.782	26.87	66.70	14.02	42
CdSe/CdS	Na_2_S, S	CuS-MoS_2_	0.62	26.25	41.22	7.03	43
CdSeTe	Na_2_S, S, NaOH	Cu_2_S	10.05	0.64	53.67	3.46	44
CdSe	Na_2_S, S, KCl	PbS/CuS	14.52	0.607	47.43	4.18	45
CdS/CdSe/ZnS	Na_2_S, S, KCl	NiCO_2_O_4_	22.49	0.574	0.43	5.55	46

a CB – carbon black, C/S – core/shell, Cu@CNR – Cu nanoparticle@carbon nanorod, NPCN – nitrogen-doped porous carbon nanoribbon.

Table 2 summarizes the parameters and performances of some electrolyte additives used in studies of QDSSCs.

**Table 2:** Solar cell parameters of QDSSCs based on polysulfide electrolyte additives^a^

QD	Electrolyte	Additive	CE	*V*_oc_	*J*_sc_	FF	*η*%	Ref.
ZCISe	Na_2_S, S, KCl	Se	Cu_2_S	0.601	26.34	0.624	9.88	47
CdS	Na_2_S, S, KCl	TEOS	Pt/CuS	0.543	19.33	0.523	5.5%	48
CdS/CdSe	Na_2_S, S	SC(NH_2_)_2_	Cu_2_S	0.50	18.98	0.36	3.42	48
ZCISe	Na_2_S, S, KCl	Se	Cu_2_S	0.601	26.34	0.624	9.88	47
ZCISe	Na_2_S, S	PEG	Cu_2_S	0.632	26.76	61.13	10. 29	15
CdSeTe	Na_2_S, S KCl	SiO_2_	Cu_2_S	710	22.21	0.712	11.23	22
CdSeTe	Na_2_S, S	PVP	Cu_2−*x*_S	0.723	20.49	0.66	9.77	21
CdSeTe	Na_2_S, S	TEOS	Cu_2_S	0.77	26.36	0.61	12.34	19
Cu–In–Se	Na_2_S thiourea	SGCN	Cu_2_S	616	21.52	53.6	7.11	18
ZCISSe	Na_2_S, S, KCl	PVP	NMC	26.52	0.802	0.720	15.31	49

a NMC – N-doped mesoporous carbon, AC– activated carbon, SGCN – sulfur-rich graphitic carbon nitride, PVP – poly(vinyl pyrrolidone), 1,5-NPTD – 1,5-naphtalenediamine, ETH – ethylamine carbon.

### 2.2 Quasi-solid-state electrolytes (gel electrolytes)

The main drawback of liquid electrolytes is their short-term stability. A liquid electrolyte evaporates easily, leaks through the electrode, has a high surface tension that reduces contact between the metal oxide and electrolyte, is volatile, flammable, difficult to handle, and is not easily filled with pore water.^50^

**Fig. 3:**
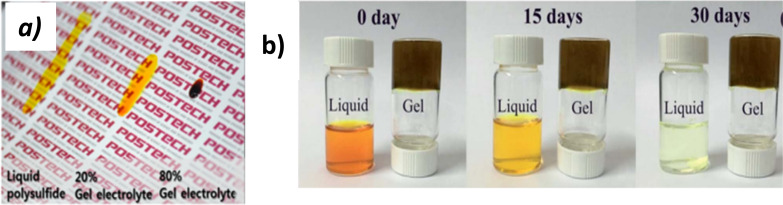
(a) Digital images of gel electrolytes with various compositions. Reprinted from ref. 57 with permission from ACS; (b) colour change of the electrolytes. Reprinted from ref. 50 with permission from the Royal Society of Chemistry, copyright 2016.

Instead of liquid-based electrolyte, quasi-solid-state electrolytes (QSSEs) may be used that contain a composite liquid electrolyte (*e.g.* polysulfide) and a solid matrix (*e.g.* polymer), whose polymer chain network is swollen by polysulfide salt solution,^51^ which may reduce the device stability of QDSSCs. In these cases, the liquid facilitates ion transport^52^ by serving as a percolation pathway and the solid matrix enhances electrolytic capability by resisting the voltage between the anode and counter electrodes. To make it easier to compare, digital images for each electrolyte as shown in Fig. 3a.

An electrolyte with a higher mechanical strength can withstand anode–cathode voltages^50,53^ while maintaining ionic conductivity.^54^

Silica nanoparticles can be used as a filler to gel and solidify the polysulfide electrolyte. The ion-adsorption centres (polar functional groups) of the polymers make silica nanoparticles a good liquid electrolyte gelling agent, which can penetrate porous TiO_2_ films and display excellent conductivity. As a thin film, it is easy to fabricate, inexpensive, and mechanically flexible.^55^ It has a low vapor pressure, excellent contact and filling properties between the nanostructured electrode and the counter electrode, good ionic conductivity, and excellent thermal stability when compared with conventional polymer electrolytes.

Liquid electrolytes are absorbed by polymers, which can improve the long-term stability of QDSSCs by preventing evaporation.^56^Fig. 3b shows there was a gradual change in colour of the liquid electrolyte over 30 days from brown to colourless, indicating that the gel polymer electrolyte was more stable than the liquid electrolyte based on these results.

**Fig. 4:**
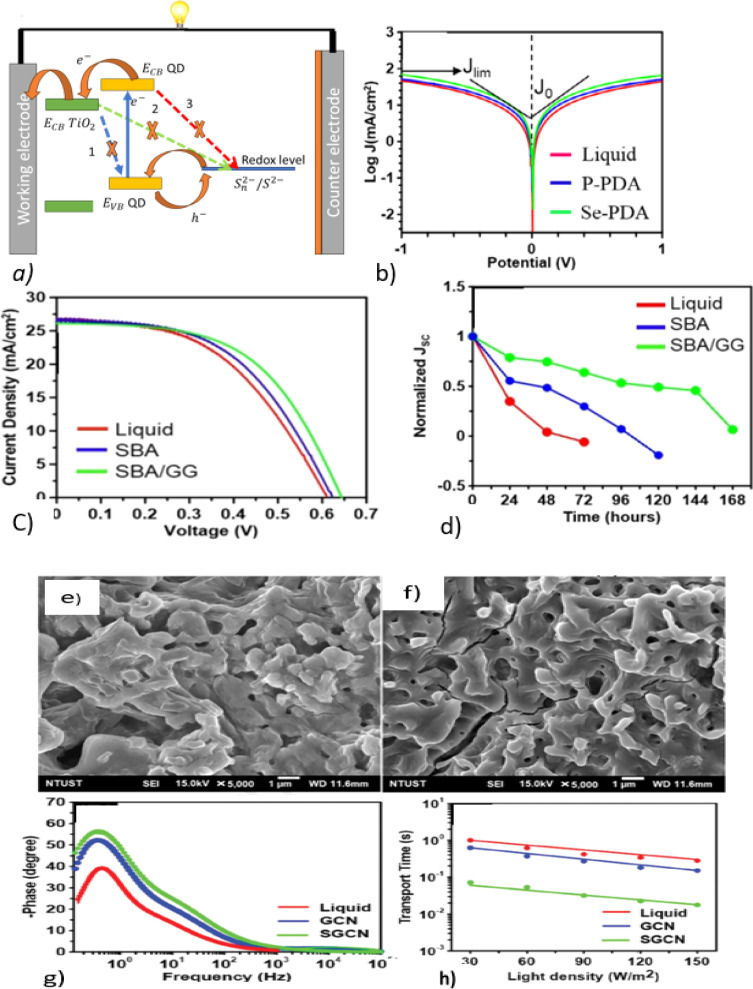
(a) Recombination pathways in XG/NP gel-electrolyte-fabricated QDSSCs; reproduced from ref. 58. (b) Tafel polarization curves; reprinted from ref. 61. (c) *I*–*V* curve, and (d) stability of a QDSSC based on a guar gum (GG) biopolymer; reprinted from ref. 60. SEM images of (e) XG gel and (f) XG/NP gel electrolytes. (g) Bode plot curves of Cu–In–S QDDSCs with different electrolytes; (h) electron-transport time obtained from IMPS and an ordered nanopores silica (SBA) gel electrolyte; adopted from ref. 58.

A QSSE based on PGE can also act as a surface passivation to reduce charge recombination, consequently enhancing the power conversion efficiency of the QSSC.^58^Fig. 4a depicts the working principles and recombination pathways in a QDSSC gel electrolyte: (1) from TiO_2_ to the QD; (2) from TiO_2_ to the electrolyte, which happens because the polysulfide species can diffuse into the TiO_2_ film and react with photogenerated electrons, resulting in electron recombination back to the electrolyte; and (3) from the QD to the electrolyte, which is due to the gel network obstructing ion movement. From Fig. 4b, it can be seen that the current density values of the co-additives (Se and PDA) are greater than that of the liquid, suggesting a faster charge-transfer kinetics at the electrolyte and electrode.

There are several disadvantages of QSSEs to note, such as structural rigidity, which makes it difficult for ions to move freely, the complex ion-diffusion pathway due to the gel-like structure, which creates a complex network for ion diffusion, and the limited solvation capabilities, which limits ion mobility. As a result, gel electrolytes have lower ionic conductivities than liquid electrolytes. The addition of ionic liquids and fillers to gel electrolytes containing many ionic carriers can help overcome these problems.^59^

Owing to their improved charge recombination from a porous structure that allows ions to freely migrate, and hydroxyl groups that form good contact with TiO_2_, the stability and PCEs of guar gum (GG) and ordered nanoporous silica (SBA)-based gel electrolytes for QDSSCs were found to be higher than those of SBA and liquid polysulfide electrolytes,^60^ as shown in Fig. 4c and d.

Rasal *et al.* added TiO_2_ to an XG-polysulfide gel electrolyte, which showed improve ionic conductivity, which in turn, increased the free volume of the polymer matrix, and thus improved the availability of ions through the growth and diffusion of the polymer.^58^ SEM images of the XG gel and XG/NP gel electrolytes are shown in Fig. 4e and f, respectively, where it can be seen that XG/NP formed a microporous framework, allowing electrolyte permeation and fast redox coupling. From Fig. 4g and h, it can be seen that the electron-transport time and recombination lifetime decreased with the increase in light intensity.

Some reported solar cell parameters based on gel electrolytes for QDSSCs are summarized in Table 4.

#### Reduction in crystallinity

An electrolyte with a high degree of crystallinity has a lower ionic conductivity. Because crystalline structures are highly oriented, their mobile carriers cannot move freely, thereby reducing the conductivity. Owing to the high secondary valence forces and the low free volume, there is a reduction in secondary valence forces, a larger free volume, better chain mobility, and thus better ionic conductivity.^62^ As a result, ionic migration in the crystalline phase is slower than in an amorphous phase.^53^ Therefore, the use of amorphous polysulfides as gelling liquid polysulfides is crucial. However, some amorphous polymers are brittle and cannot withstand the electric current between the anode and the cathode. Electrolytes based on polymer blends exhibit excellent mechanical, thermal, and electrical properties.^63^

#### Reduction of ion-pair formation

Electrolytes with ion pairing have reduced ionic conductivity.^64^ In solution, ionic conductivity occurs when free ions carry electrical current. During ion-pair formation, the number of free ions decreases, which decreases the ionic conductivity of the electrolyte. As the ion concentration increases, more ionic compounds are dissolved into free ions in the solvent, which increases the ionic conductivity.^65^

### Improving the temperature of the electrolyte

**Fig. 5:**
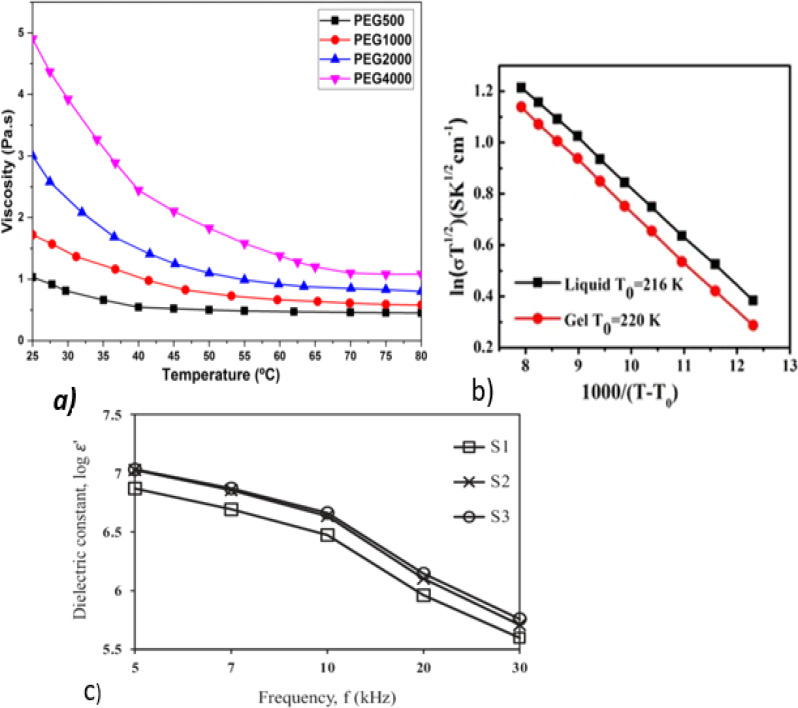
(a) Viscosity as a function of temperature; reproduced from ref. 67 with permission from Springer Nature. (b) Temperature dependence of the ionic conductivity of GE electrolytes; adopted from ref. 69. (c) Dielectric constant as a function of frequency.^66^

Polymer chains become more mobile as the electrolyte temperature increases because the electrolytes are more mobile, molecular distances are larger, secondary valence forces are smaller, and the ionic conduction resistance decreases.^62^Fig. 5a and b show the temperature-dependent ionic conductivity of a gel electrolyte. The temperature dependence of the electrolytes can be calculated using the Arrhenius equation:^51^3
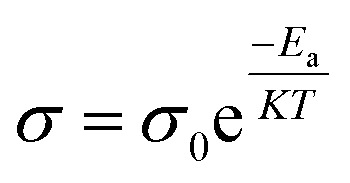
where, *σ* is conductivity in siemens per meter (S m^−1^), *σ*_0_ is material constant, *K* is Boltzmann's constant, which is 1.3806 × 10^−22^ joules per kelvin, *T* is the temperature in kelvin, “*E*_a_” is the activation energy in joules.

#### Dielectric constant

The ionic conductivity of an electrolyte can depend on the dielectric constant. The dielectric constant of an electrolyte measures its ability to separate charge. The dielectric constant of an electrolyte affects its ionic conductivity. Lowering the electrostatic interactions improves the ionic conductivity and mobility. By using solvents with high dielectric constants, ions can be more easily dissolved and their interactions diminish. As a result, the electrolyte's ionic conductivity and mobility are improved as a result. By decreasing the coulombic interactions between molecules, a greater dielectric constant indicates a higher ionic mobility and ionic conductivity. A higher dielectric permittivity, mobility, and charge carrier density lead to higher ionic conductivity, as shown in Fig. 5c.^66^ Siddhant B. Patel and Jignasa V. Gohel added Li-TFSI to a GPE, which improved the ionic conductivity and reduced the internal resistance of the devices, allowing them to display a superior PCE of 4.19%.^67^

#### Doping of the electrolyte

GPE doping enhances the charge carriers, reduces the crystallites, and increases the amorphocity.^68^ Amorphous materials have rich ion-conduction properties.

Research on polyelectrolytes has recently focused on those with polar functional groups, superior adsorption, and a water holding capacity, making them suitable electrolyte hosts for QDSSCs.^70^

Prakash and coworkers used graphene oxide (GO) to functionalize polyurethane (FPU). For the QDSSC gel electrolyte, they used co-sensitized CdS/CdSe as a sensitizer, resulting in a PCE of 1.71%. This may be due to the higher conductivity and reduced recombination. Polyurethanes represent a group of polyelectrolytes comprising hard di-isocyanate and chain extenders that provide strength, hardness, and high-temperature performance, as well as soft segments of polyether or polyester polyol, which provide flexibility, toughness, and resilience.^71^ The chain on a hard segment is used to increase the chain length and molecular weight, thereby reducing crystallization. The functionalization of polyurethane provides additional active pendant anion on the hard segment content.^70^ Using MPA-capped CdS as a sensitizer and FTO/rGO/TiO_2_ as a photoanode, Kumar and coworkers functionalized PU with graphene, a carboxylate, and a sulfonate segment as pendant anions (FTO/rGO/TiO_2_ photoanode and FTO/Pt counter electrode), exhibiting power conversion.^70^ As an electrolyte for a CdS quantum dot sensitizer, Ravi, and Maiti functionalized polyurethane using 3-mercapto propionic acid.^72^ Kumar and coworkers, in another work, used PU as a gel electrolyte in QDSSCs with TiO_2_/CdS photoanodes and achieved a PCE of 1.25%.^73^

Table 3 lists shows some polyelectrolytes gel electrolytes used with QDSSCs.

**Table 3:** Solar cell parameters of some reported QDSSCs based on polyelectrolytes^a^

CE	QD	Electrolyte	*V*_oc_	*I*_sc_	FF	PC	Ref.
Pt	CdS/CdSe	PU	4.66	0.74	0.44	1.51	72
Pt	CdS	PANi	2.2	0.60	0.78	1.25	73
Pt	CdS/CdSe/ZnSe	SPU-GO-CC	0.73	5.09	0.46	1.71	20
Pt	CdS	PUI-GO	0.594	6.44	0.43	1.63	70
Pt	CdS	S(PU + CB)-PCL-EDA	0.51	5.95	0.38	1.16	74

a gPU – polyurethane, PANi – polyaniline, PUI-GO – functionalized polyurethane ionomers-graphene oxide, SPU-GO-CC – sulfonation of polyurethane graphene oxide conductive carbon, S(PU + CB)-PCL-EDA – sulfonic, polyurethane, carbon black, ethylene diamine, and PCL composite ionomer.

**Table 4:** Solar cell parameters for some reported QDSSCs based on gel electrolytes^a^

CE	El. comp	Gelator	QD	*V*_oc_	*I*_sc_	FF	*η*%	Ref.
CoSe	Na_2_S, S	PAAm/GO	CdS/CdSe	0.513	12.18	65.6	4.10	56
Cu_2−*x*_S	Na_2_S, S	CMS-Na	CdSe	0.615	15.63	65.8	6.32	69
Pt	Na_2_S, S	PAAm-PAA	CdS	14.77	0.37	0.44	2.24	66
CZTS/Pt	Na_2_S, S	PEG	CdS	0.59	11.47	60.5	4.09	50
Pt	Na_2_S, S	PAAm-G	CdS	0.590	9.63	39.4	2.24	75
Cu_2_S	Na_2_S, S	PAM-MBA	CdS/CdSe	534	12.4	0.601	4.0	11
Pt	Na_2_S, S	Dextran	CdS/CdSe	399	2.45	0.56	4.58	76
Cu_2_S	Na_2_S, S	KGM	CdS/CdSe	12.76	503	0.63	4.06	77
Pt	Na_2_S, S	12-HDXA	CdS/CdSe	0.47	12.18	0.42	2.40	78
CuS	Na_2_S, S	Se-PDA	Cu–In–S	659	23.15	56.3	8.59	61
Cu_2_S/RGO	Na_2_S, S	Agar	CdS	0.578	14.13	0.39	3.09	2
Cu_2−*x*_S	Na_2_S, S	PVP	CdSeTe	0.723	20.49	0.66	9.77	21
CuS	Na_2_S, S, KCl	XG/TiO_2_	Cu–In–Se	667	22.9	53.7	8.19	58
Pt	Na_2_S, S	PbS-CnF	CdS	446.1	9.95	34.75	1.51	79
Pt	Na_2_S, S, KCl	A-SiO_2_	CdS/CdSe	625	18.95	0.6	7.11	24
Cu@CNR	Na_2_S, S, KCl	CM-Na	ZCISe	0.628	26.46	0.59	9.50	34

a 12-HDXA – 12-hydroxystearic acid, PVP – poly(vinyl pyrrolidone), PEG – polyethylene glycol, XG/NP – TiO_2_ incorporated into xanthan gum, SBA/GG – nanoporous silica (SBA) incorporated into guar gum (GG), PbS-CnF – lead quantum dot cellulose acetate nanofiber, A-SiO_2_ – NH_2_-rich silica nanoparticle, Se-PDA – Se-doped polydopamine, KGM – Konjac glucomannan, PAM–MBA – polyacrylamide-bis-acrylamide, PAAm-G – graphene-implanted polyacrylamide, PAAm/GO – graphene oxide-tailored polyacrylamide, CMS-Na – sodium carboxymethyl starch, PAAm-PAA – poly(acrylamide-*co*-acrylic acid).

### 2.3 Solid-state electrolyte for QDSSCs

QSSE are limited by the leakage of organic solvents and liquid parts from the polymer host, which affects the stability, ionic conductivity, and electrode performance.^80^ Rather than QSSEs or liquid electrolytes, researchers have focused on solid electrolytes, which are simple to manufacture and can improve the stability of the devices. In contrast, these have poor permeability and low ion mobilization, which results in low ionic conductivity, and poor penetration into the nanostructure,^81^ which results in a low power conversion efficiency compared to liquids and gels.

In solid-state electrolytes, the electrons cannot move quickly. Rapid electron mobilization leads to higher ionic conductivity, which facilitates the redox cycle and accelerates, the injection of electrons from quantum dots to TiO_2_. These factors affect the difference between the Fermi level of electrons in TiO_2_ and the redox potential of the electrolyte, or the open circuit voltage.^82^ These factors improve the ionic conductivity of the electrolyte, improving the generation of photocurrents and the overall PCE.

**Fig. 6:**
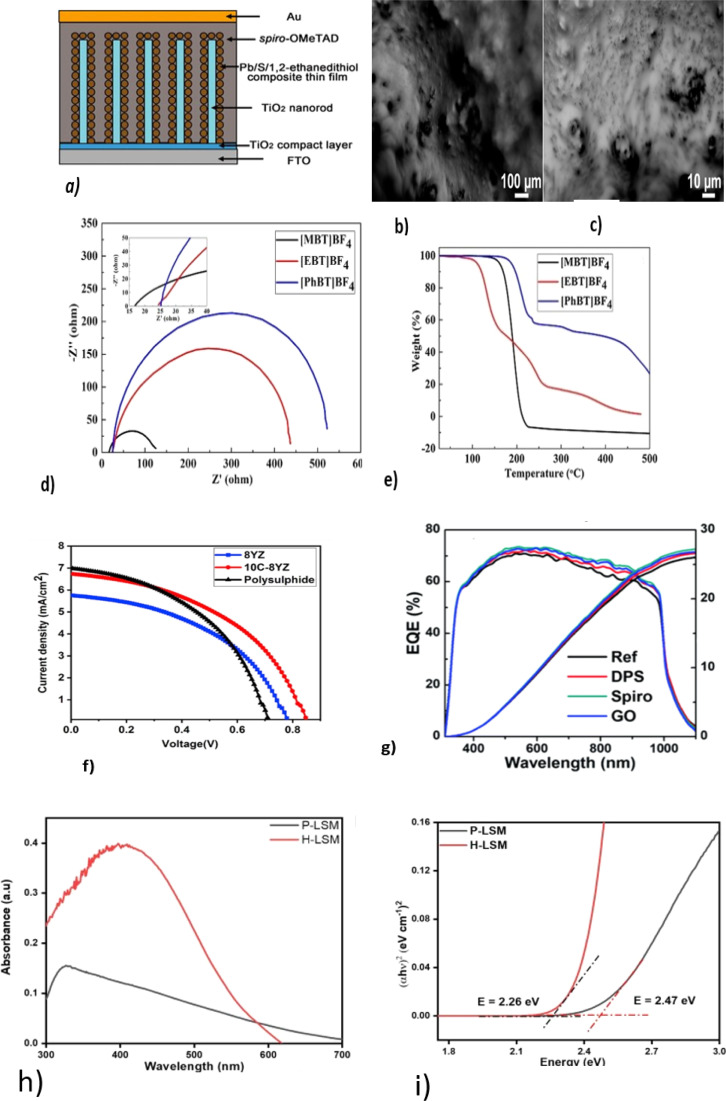
(a)Schematic diagram of Pb/S/1,2-ethanedithiol composite thin-film-sensitized TiO_2_ nanorod array solar cells; reproduced from ref. 83 with permission from Springer Nature copyright 2018. (b and c) SEM image of a solid electrolyte at low and high magnifications; reproduced with permission from the American Chemical Society, copyright 2019.^88^ (d) Nyquist plots of [DHexBIm][X] (X = Br, BF_4_ and SCN)-based electrolytes; reproduced from ref. 89, with permission from the Royal Society of Chemistry, copyright 2019. (e) TGA curves of OICs [MBT]BF_4_, [EBT]BF_4_ and [PhBT]BF_4_; reproduced from ref. 90. (f) *J*–*V* curves for the Pechini method and hydrothermal method. (g) EQ and integrated *J*_sc_ curves; reprinted from ref. 85. (h) UV-visible spectra and (i) Tauc plots of H-LSM and P-LSM to determine the optical bandgap; reproduced from ref. 97.

Ma. Chengfeng *et al.* developed a solid-state electrolyte from spiro-OMeTAD for QDSSCs using Pb/S/1,2-ethanedithiol QD as an absorber onto TiO_2_ nanorods, as shown in Fig. 6a, with a PCE of 4.51%.^83^ Spiro-OMeTAD is mostly used as a solid-state electrolyte in QDSSCs because of its high ionic conductivity, high stability, and compatibility with other device structures.^84^

According to Yu Lin and colleagues,^85^ the quantum yield (EQE) of 2,2,7,7-tetrakis[*N*,*N*-di(4-methoxyphenyl)-amino]-9,9-spiro-bifluorene-based HTMs is higher than that of graphene oxide (GO), and diphenyl sulfide (DPS), as shown in (Fig. 6g). Based on this, it can be concluded that spiro-based solid-state electrolytes (HTM) can improve the overall stability and *V*_oc_ of devices more than DPS and GO. The higher the EQE, the better the long-term stability, possibly due to more photons being absorbed, which reduces the stress on materials and interfaces. As a result, device degradation based on HTMs may be improved.^86^

There are several strategies to deal with ionic conductivity issues in solid-state electrolytes, including the use of plastic crystals, such as succinonitrile polymers and ammonium salts. Succinitrile polymers are the plastic crystalline phases that can be used as additives in polymer electrolytes to improve their ionic conductivity of electrolytes.^87^ These plasticizers are used to improve electrolyte leakage and decrease device degradation through resisting corrosion of the device by the absorption of liquid polysulfide on the counter electrode, resulting in a good compatibility of the electrolyte with the sensitizer.

Kokal, Ramesh K. *et al.* developed a solid-state electrolyte based on succinonitrile/Na_2_S for a CdS QDSSC with a maximum PCE of 6.3%, *V*_oc_ of 753 V, *J*_sc_ of 13 mA cm^−2^, and FF of 61.^88^ According to their work, solid-state electrolytes have increased thermal, electrical, and ionic conductivity. Raising the electrolyte temperature increases the electron density and electron mobility. In turn, this improves the performance of the device. Fig. 6(b and c) depict SEM images at low and high magnifications for succinonitrile and Na_2_S solid electrolytes. There has been research done to enable solid-state electrolytes to have good conductivity and a good contact between the electrolyte and the electrodes.^89^ By substituting benzothiophenium with S. Wang and coworkers developed a solid-state electrolyte that co-sensitized CdS/CdSe quantum dots. As a result of the saturated carbon chain, they were able to obtain the highest optical absorption and ionic conductivity from *S*-methyl benzothiophenium tetrafluoroborate ([MBT]BF_4_) with a voltage of 0.71 V and PCE of 5.49% (ref. 90) (Fig. 6d). From the TGA results, it was found that ([MBT]BF_4_) had good thermal stability at an operating temperature of 100 °C, as depicted in Fig. 6e.

It is possible to improve the adsorption and diffusion of electrolytes by using oxide nanoceramics, which are inexpensive, easy to process, and can form highly porous solid-state electrolytes.

Kusuma and colleagues developed solid-state electrolytes for QDSSCs using yttria-stabilized zirconia and ceria instead of polysulfides. Ceramic dopants can be used to create oxygen vacancies that result in band shifting, reduced electron recombination, and increased device stability. Using a ceramic oxide, the stability of one device lasted 60 days, whereas with polysulfide it lasted only 5 days.^91^

As reported by Akhil S. *et al.*, it is possible to increase the stability of a device by using a perovskite material in a solid electrolyte. This might be because perovskite materials are chemically stable and can resist light, heat, and moisture, which could otherwise degrade and reduce the stability of devices. In addition they have high ion conductivity, therefore they are ideal solid electrolytes for QDSSCs.^92^ Moreover perovskite materials can be used in conjunction with other components of the devices, such as counter electrodes and photoanodes.^93^

The synthesis method can affect the optical properties of perovskite-based solid-state electrolytes. Akhil and coworkers used hydrothermal (HLSM) and Pechini (PLSM) methods to synthesize a solid-state electrolyte for a QDSSC with an improved PCE of 1.73%. An electrolyte containing ceramic particles generally improves ionic conductivity at high temperatures, allowing efficient ion transport to improve the power conversion efficiency, as shown in Fig. 6f. Using HLSM, a lower band gap and higher absorption maxima of 2.26 eV and 410 nm, respectively, were obtained, as shown in Fig. 6(h and i), indicating that the hydrothermal synthesis of perovskites enables better charge-transport properties at the electrolyte/electrode interface. Hydrothermal synthesis can produce smoother, more uniform surfaces, a slower growth rate, more controlled nucleation and growth of the crystal, and lower defects^94^ than the Pechini method. This results in a narrower band gap and high UV-visible absorption. In addition, surface and interface effects can contribute to the observed higher UV-visible peak in the absorbance spectrum of hydrothermally synthesized^95^ perovskites.

Quantum dot-sensitized solar cells based on solid-state electrolytes can perform better with an increased quantum dot loading amount,^96^ which can provide more number active sites, doping to optimize the interfacial band alignment, and insertion of a passivation layer to reduce the charge-recombination resistance and increase the PCE. In addition, several plastic crystalline and p-type semiconductors have received attention because of their low cost, high stability, and high-performance solid-state hole-transport properties. In Table 5, the parameters of some quantum dot-sensitized solar cells fabricated using solid-state electrolytes are summarized, showing that QDSSCs with solid-state electrolytes currently still have lower efficiency.

**Table 5:** Solar cells parameters based on solid-state electrolytes used in QDSSCs^a^

Electrolyte	QD	CE	*V*_oc_	*J*_sc_	FF	PCE	Ref
PVP/Na_2_S–S	CdS	CoSe	0.67	2.84	28.9	0.55	98
[MBT]BF_4_	CdS/CdSe	PbSe	0.71	20.73	37.30-	5.49	90
NaCMC	CdS	Pt	0.42	5.27	0.50	0.96	82
succinonitrile/Na_2_S	CdS	CoSe	0.670	3.65	52.7	1.29	99
Spiro-OMeTAD	(Pb,Cd)S	Au	0.49	1.79	0.36	0.32	100
Spiro-OMeTAD	PbS	Au	0.56	13.00	64.8	4.75	101
Spiro-OMeTAD	CuInS_2_	Au	0.48	4.21	0.37	0.75	96
Spiro-OMeTAD	CuInS_2_	Ag	0.64	5.34	0.41	1.41	102
NaCMC	CdS/ZnS	Pt	5.27	0.45	0.50	0.96	82
Succinonitrile	CdS/ZnS	C	753	13	61	6	88
Spiro-OMeTAD	CIS	Au	0.68	11.33	0.41	3.13	96
Spiro-OMeTAD	Pb/S/1,2-ethanedithiol	Au	0.56	11.84	0.68	4.51	83
Spiro-OMeTAD	PbS	Au	0.49	14.47	59.65	4.25	101
Spiro-OMeTAD	Sb	Ag	9.84	0.523	0.600	3.09	103
P3HT	PbS[CuS]	Au	0.6	20.7	65	8.07	104
Spiro-OMeTAD	PbS	Au	0.52	13.56	57.88	4.10	105
P3HT	PbS/CuS	Au	0.58	20.1	60.5	7.0	106

a PVP/Na_2_S–S – polyvinylpyrrolidone/polysulfide, spiro-OMeTAD – 9,9′-spirobifluoride, [MBT]BF_4_-S – methyl benzthiophenium tetrafluoroborate, NaCMC – sodium-carboxymethylcellulose, (P3HT) – poly(3-hexylthiophene).

## 3. Future prospects and conclusion

In this review, we examined quantum dot-sensitized solar cells in terms of their current state and future prospects, especially regarding the electrolyte stability. We focused the reader's attention on the state-of-the-art efforts and advancements in improving the stability and performance of QDSSCs that have been occurred over the last decade, which have promoted their unique properties, like facile fabrication, low cost, tuneable bandgap, and generation of multiple excitons, and that have continually sought to increase their power conversion efficiency. Their potential is recognized as a part of all future energy technologies. A variety of electrolytic liquids, gels, and solid electrolytes have been used to enhance the PCE and stability. The PCE of QDSSCs has now reached over 15%. The electrolytes need to be modified for improving the QDSSC stability and PCE even more. Liquid electrolytes have a higher power conversion efficiency and are less stable than quasi-solid-state electrolytes and solid electrolytes. QDSSC needs to have a high power conversion and stability to be commercially successful. Solid-state QDSSCs are expected to be a promising direction for solving the stability problem of QDSSCs. Liquid junction devices are susceptible to electrolyte leakage and anodic corrosion. Several polymers are used to gel and solidify polysulfide electrolytes in QDSSCs. However, their power conversion is still lower than that of liquid electrolytes. Adding redox couples with low redox potentials is necessary to improve the efficiency of QDSSCs.

## Author contributions

Bayisa Batu Kasaye: conceptualization and writing – original draft. Megersa Wodajo Shura: conceptualization, supervision and editing. Solomon Tiruneh Dibaba: provisioning of resources.

## Conflicts of interest

There are no conflicts to declare.

## Supplementary Material
